# A Treatise on Compound Ophthalmic Lenses

**Published:** 1887-04

**Authors:** 


A TREATISE ON SIMPLE AND COMPOUND OPHTHALMIC LENSES, their refraction and dioptric formulæ, including tables of crossed cylinders and their sphero-cylindrical equivalents. By Chas. F. Prentice. 8vo. cloth, $1.50. Published by James Prentice & Son, Opticians, 178 Broadway, N. Y.

The number of new and original illustrations, by the author, contained in this work exhibits clearly the various forms of compound lenses and the character of their refraction for parallel rays, including those of plano-convex and concave lenses of compound astigmatic refraction, thus making the treatise a clear and instructive work.

